# Supplementary material for: Chromothripsis during telomere crisis is independent of NHEJ, and consistent with a replicative origin
Source: Genome Res. 2019 May;29(5):737–49. doi: 10.1101/gr.240705.118 (PMC6499312; doi:10.1101/gr.240705.118)
Supplement: Supplemental Material [file supp_gr.240705.118_Supplemental_file_1.zip › contigs/annotated_contigs/DB70/contig.2.DB70_length_266_mean_cov_3.38345864662.docx]

**DB70_length_266_mean_cov_3.38345864662**

```
TAGTGCTAAATTTGAAGACCCTTAAAGGTAAGGAACATGTATAATTCAGCATATTGTTTTTCAGACCCCATCATAAGGCATAGTACATA
 >chr13:70530372-70530578 + E=4e-113
GTAGACATACATATAGTAAAACTTGAGAGTTGCTCAATGCATTCACAGGACAAAGACCATTAGGCATTAATATGCTTAAATTGTGAGGC

ACCATTGACACCTAAAGGGAACCAGCAT|CCTCATGCTTCTGAGGATGACGCATGCAATAACTCCTGAATAACTTTAACTTCACCCTGC                              >chr13:70531151-70531211 + E=8e-25
```
